# Effect of preoperative prophylactic intravenous tranexamic acid on perioperative blood loss control in patients undergoing cesarean delivery: a systematic review and meta-analysis

**DOI:** 10.1186/s12884-023-05753-9

**Published:** 2023-06-06

**Authors:** Fan Yang, Han Wang, Mengdie Shen

**Affiliations:** 1 Department of Intensive Care Unit, Women’s Hospital, School of Medicine, Zhejiang University, Zhejiang Province, Hangzhou, 310003 People’s Republic of China; 2 Department of Gynecology and Obstetrics, Women’s Hospital, School of Medicine, Zhejiang University, Zhejiang Province, Hangzhou, 310003 People’s Republic of China; 3 Department of Internal Medicine, Women’s Hospital, School of Medicine, Zhejiang University, Zhejiang Province, Hangzhou, 310003 People’s Republic of China

**Keywords:** Tranexamic acid, Postpartum hemorrhage, Cesarean delivery

## Abstract

**Background:**

Postpartum hemorrhage (PPH) is one of the important risk factors leading to maternal mortality and intervention is essential. Oxytocin therapy is widely used clinically, but the effect is unsatisfactory. The efficacy of tranexamic acid (TXA) in hemostasis is notable, whereas its use in preventing PPH warrants exploration.

**Aims:**

To evaluate the effect of prophylactic administration of TXA on perioperative blood loss in women undergoing cesarean section by systematic review and meta-analysis of published studies.

**Methods:**

Bibliographic databases were screened from their inception to December 2022 to retrieve relevant studies. Study outcomes including blood loss during cesarean section, 2-h postpartum blood loss, total blood loss (during cesarean section and 2-h postpartum), and 6-h postpartum, as well as hemoglobin changes were extracted and compared.

**Results:**

A total of 21 studies, nine randomized clinical trials and 12 cohort studies, involving 1896 patients given TXA prophylactically and 1909 patients given placebo or no treatment, were analyzed. Compared with the control group, the preoperative prophylactic intravenous administration of TXA significantly reduced the intraoperative (RCT: *P* < 0.00001, cohort studies: *P* < 0.00001), 2-h postpartum (RCT: *P* = 0.02, cohort studies: *P* < 0.00001) and total blood loss (RCT: *P* < 0.00001, cohort studies: *P* = 0.0002), and reduced the decline in hemoglobin (RCT: *P* < 0.00001, cohort studies: *P* = 0.0001), but did not significantly affect blood loss at 6-h postpartum (*P* = 0.05).

**Conclusion:**

Prophylactic intravenous TXA before cesarean section is helpful in preventing perioperative bleeding in women.

**Trial registration:**

http://www.crd.york.ac.uk/PROSPERO, identifier: CRD 42022363450.

## Introduction

Postpartum hemorrhage (PPH) is a serious complication during delivery as it can lead to maternal death and severely impacts families and society. Worldwide, approximately 530,000 women die from pregnancy complications every year, of which less developed countries account for the majority [1]. The incidence rate of PPH in developed countries is also on the rise. According to the American College of Obstetricians and Gynecologists (ACOG) data [2], postpartum hemorrhage causes approximately 11% of maternal deaths in the United States. Although there are some identifiable high-risk factors for PPH, many cases occur suddenly. There has been significant progress in medical care around the world in the twenty-first century. The current standard of care for PPH is postpartum administration of uterotonic agents, which promotes uterine contraction and reduces vaginal bleeding in both vaginal delivery and cesarean section [3]. However, high-concentration uterotonic agents cannot be given prior to delivery. Therefore, early intervention and alternative therapeutic options are essential for PPH to effectively reduce the risk of death and improve maternal outcomes.

Tranexamic acid (TXA) is a synthetic antifibrinolytic drug and inhibits the interaction between fibrinolysin and fibrin to stabilize the fibrin matrix [4]. It is effective in preventing bleeding complications and improving the clinical outcomes of patients with trauma [5]. In the past decades, many studies [6, 7] have shown that TXA can effectively reduce surgical blood loss and blood transfusion requirements. In 2022, Devereaux et al. [8], conducted a trial in which 9535 patients undergoing noncardiac surgery were randomized them to TXA or placebo. TXA administered patients showed significantly lower incidence of major organ bleeding, with no significant increase in thrombosis related adverse outcomes. TXA was found to be effective in reducing blood loss in multiple types of surgery, including gynecological related procedures. However, currently there are no clear recommendations for the preoperative application of TXA to prevent intraoperative bleeding. Although the WOMAN trial [9] showed that the administration of TXA was effective in the treatment of PPH, reducing mortality by 20–30%, recommendations for the application of TXA to prevent PPH before the start of cesarean section has not been developed. Therefore, in this study we performed a meta-analysis of preventative TXA administration on the effect of maternal blood loss by cesarean section to generate addition evidence for the use of this drug for PPH.

## Materials and methods

We performed this systematic review and meta-analysis using predefined protocols and reported methods according to the statement of the Preferred Reporting Items for Systematic Reviews and Meta-Analyses (PRISMA) [10]. This research protocol has been prospectively registered and can be obtained online. (PROSPERO registration number: CRD 42022363450).

### Search strategy

Literature searches of the following databases: Medline, PubMed, Web of Science, Embase, and the Cochrane Library, were systematically performed from their inception to December 2022. The lists of included articles were screened to determine potentially missed literatures and relevant studies were screened as completely as possible. We used the following MeSH (Medical Subject Headings) terms alone or in combination for article retrieval: "postpartum hemorrhage", "tranexamic acid" and "cesarean section". The database search was performed on 28^th^ September 2022, and then updated on 26^th^ December 2022.

### Inclusion and exclusion criteria

Studies with the following criteria were included: (1) patients: pregnant women who were scheduled to undergo cesarean section; (2) interventions: TXA administered prior to the start of surgery. Comparator: placebo or no treatment before the start of surgery. Oxytocin was prescribed as prophylaxis for bleeding in both groups; (3) Outcomes: the primary outcome was postpartum blood loss, including intraoperative, 2-h postpartum, and total (intraoperative and 2-h postpartum). Secondary outcomes were hemoglobin changes, and 6-h postpartum blood loss. Exclusion criteria: (1) patients who did not undergo cesarean delivery; (2) patients who were not treated with oxytocin; (3) deficiency of control groups and information of the primary outcomes.

### Study selection and data extraction

In this study, two independent researchers (FY and HW) searched each database separately, screened articles according to the titles and abstracts, excluded articles unrelated to this study, and determined articles that conform to the criteria. Subsequently, the full texts of articles that met the inclusion criteria were obtained, and information relating to population, medications used, postpartum blood loss, hemoglobin changes characteristics were extracted. For articles where information was not fully available, we tried to contact the original authors and endeavored to compensate for missing information. Any disagreements among the two study researchers were resolved with mutual discussion, or a third researcher (MS) was asked to assist with the screening and evaluation.

### Quality assessment

We used the Cochrane Collaboration’s tool [11] to evaluate the risk of bias in randomized control trial (RCT) studies and the Newcastle Ottawa scale (NOS) [12] was employed to evaluate the cohort studies. The Cochrane risk assessment tool is based on a point-by-point review of each included study and assess seven types of biases: random sequence generation (selection bias); allocation concealment (selection bias); blinding of participants and personnel (performance bias); blinding of outcome assessment (detection bias); incomplete outcome data (attrition bias); selective reporting (reporting bias); other bias. Studies were classified as low, unclear, or high risk for each bias category. The NOS is a quality assessment tool used for observational studies. For cohort studies, the quality levels of three areas: group comparability, study group selection, and outcomes were assessed. High quality studies are those that score five points or above out of a total score of nine points. The assessment was performed independently by two reviewers to ensure accuracy. Any disagreements among the two study researchers were resolved with mutual discussion, or a third researcher was asked to assist with the screening and evaluation.

### Sensitivity analysis

We used the method of leave-one-out to perform sensitivity analysis for RCT studies and cohort studies separately. We examined whether the results substantially changed by removing the data from one study each time from the included studies for further analysis.

### Statistical analysis

The Revman software (version 5.3.0, Copenhagen, Denmark) was used for meta-analysis. We tested the included studies for heterogeneity by evaluating the *I*^*2*^ statistic. *P* > 0.1 and *I*^*2*^ ≤ 50% are indicators of acceptable heterogeneity level and allows the use of a fixed-effect model. The random-effect model was used when *P* ≤ 0.1 and *I*^*2*^ > 50%. The *P* value of the combined statistics was generated with *P* ≤ 0.05 considered as statistically significant [13, 14]. For continuous parameters (blood loss, hemoglobin change), we calculated the mean difference (MD) between the two groups.

## Results

**Fig. 1:**
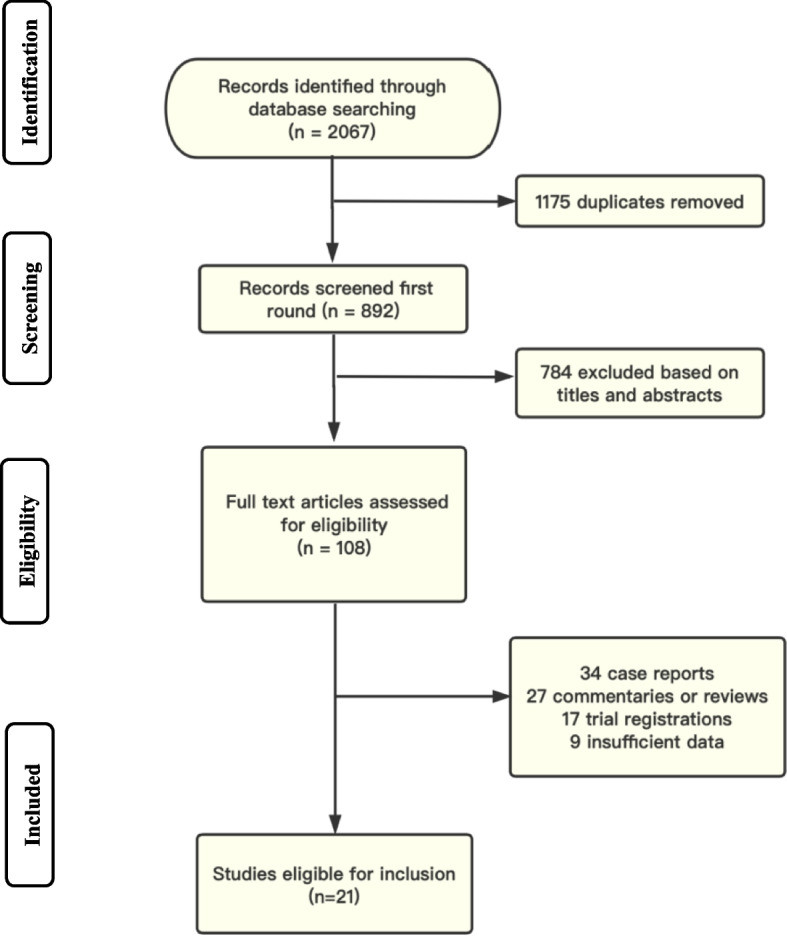
Study flow diagram. Records were identified through database searches and grey literature. A total of 108 articles met the criteria for full-text review, and 21 of them were finally included in the meta-analysis

Through a systematic search, a total of 2067 studies were screened. Based on titles and abstracts, 108 studies were selected for full-text review. Finally, 21 studies (9 RCT; 12 cohort studies) including 1896 patients given TXA prophylactically and 1909 patients given placebo or no treatment, met the inclusion criteria for this study (Fig. 1).

### Study characteristics

There were 2295 patients in the nine RCT studies, of which 1140 patients received prophylactic TXA and 1155 received placebo or no treatment. In the TXA group, eight studies used 1 g of TXA while one study used 10 mg/kg. In the control group, seven studies used placebo and two had no treatment. In the twelve cohort studies there were a total of 1510 patients, of which 756 received prophylactic TXA and 754 were administered placebo or no treatment. In the TXA group, nine studies used 1 g of TXA, and three studies used 10 mg/kg. In the control group, five studies used placebo while seven did not use any treatments. Details of all 21 studies are summarized in Tables 1, 2 and 3.

**Table 1:** Summary of included studies

Randomized Controlled Trial (RCT)						
**Author**	**Year**	**Country**	**Patients (n)** ^a^	**Intervention, TXA**	**Control**	**Uterotonic standard prophylaxis**
Ali Movafegh [15]	2011	Iran	100 (50/50)	10 mg/kg TXA in 200 mL of normal saline, IV. 20 min before beginning spinal anesthesia, over 10 min	200 mL of normal saline, IV. 20 min before beginning spinal anesthesia, over 10 min	10 IU oxytocin in 500 mL of normal saline IV, after delivery of the placenta, over 20 min
H. Abdel-Aleem [16]	2013	Egypt	740 (373/367)	1 g/10 mL TXA diluted with 20 mL of 5% glucose, IV. before the operation commenced, over 10 min	No treatment	5 IU oxytocin IV bolus and 20 IU oxytocin intravenous infusion
Mehmet B. Sentu ¨rk [17]	2013	Turkey	223 (101/122)	1 g of TXA diluted with 20 mL of 5% glucose, IV. 10 min before skin incision, over 5 min	20 mL of 5% glucose, IV. 10 min before skin incision, over 5 min	20 IU oxytocin IV in bolus form after removal of placenta
Anuradha Ghosh [18]	2014	India	140 (70/70)	1 g/10 mL TXA, IV. before skin incision	10 mL of sterile water, IV. before skin incision	20 IU oxytocin during the first 8 h post operatively
Haitham Torky [19]	2021	Egypt	120 (60/60)	1 g/10 mL TXA, IV. 20 min before the procedure	10 mL normal saline, IV. 20 min before the procedure	10 IU oxytocin IM + 10 IU oxytocin IV, immediately after delivery
Abd El-Naser Abd El-Gaber [20]	2019	Egypt	500 (250/250)	1 g of TXA, IV. 10 min before skin incision, over 2 min	normal saline, IV. 10 min before skin incision, over 2 min	10 IU oxytocin in 500 mL of normal saline IV, after the delivery of baby, over 15 min
Forozan Milani [21]	2019	Iran	60 (30/30)	1 g of TXA diluted in 20 mL of 5% distilled water, IV. 15 min before skin incision. 15 min before skin incision	10 mL distilled water diluted in 20 mL of 5% distilled water, IV. 15 min before skin incision	30 IU oxytocin in 1L ringer lactate IV, after the delivery
Zahra Naeiji [22]	2021	Iran	200 (100/100)	1 g/10 mL (body weight < 90 kg) or 1.5 g/15 mL (body weight > 90 kg) diluted in 15 mL of 5% dextrose, IV. just before skin incision	5 mL of distilled water in 15 mL of 5% dextrose, IV. just before skin incision	20 IU oxytocin in 500 mL normal saline, IV, 8 mU/min, after delivery
Amr H. Yehia [23]	2014	Egypt	212 (106/106)	1 g/10 mL TXA, IV. with induction of anesthesia, over 2 min	No treatment	10 IU oxytocin, IV. after delivery of the baby
**Cohort Studies**						
Upasana Goswami [24]	2013	India	60 (30/30)	10 mg/kg TXA in 20 mL of 5% dextrose, IV. 20 min before skin incision	5 mL of distilled water in 20 mL of 5% dextrose, IV. 20 min before skin incision	20 IU oxytocin in 500 mL normal saline, IV, 8 mU/min, after delivery of the neonate
Ming-ying Gai [25]	2004	China	180 (91/89)	1 g/10 mL TXA diluted with 20 mL of 5% glucose, IV. 10 min before incision, over 5 min	No treatment	10 IU oxytocin IV + 20 IU oxytocin into the intra-uterine wall after delivery of the neonate
Tullika Singh [26]	2014	India	200 (100/100)	1 g of TXA, slowly IV. 20 min before skin incision, over 5 min	No treatment	20 IU oxytocin in 500 mL of ringer lactate, after delivery of baby
A. V. Chandak [27]	2017	India	100 (50/50)	1 g of TXA, slowly IV. 20 min before skin incision, over 5 min	No treatment	20 IU oxytocin in 500 mL normal saline, IV, 8 mU/min, after delivery of the neonate
Leila Sekhavat[28]	2009	Iran	90 (45/45)	1 g/10 mL TXA, IV. 10 min before incision, over 5 min	10 mL of 5% glucose, slowly IV. 10 min before incision, over 5 min	10 IU oxytocin in 500 mL dextrose normal saline, over 30 min, after delivery
Irene Ray [29]	2016	India	100 (50/50)	1 g/10 mL TXA in 20 mL of 5% dextrose solution, IV. 20 min before beginning of spinal anesthesia	30 mL of 5% dextrose solution, IV. 20 min before beginning of spinal anesthesia	10 IU oxytocin in 500 mL of normal saline IV, over 20–30 min, after delivery
S.J. Dhivya Lakshmi [30]	2016	India	120 (60/60)	1 g/10 mL TXA in 100 mL of normal saline, IV. at least 20 min before skin incision, over 15 min	No treatment	10 IU oxytocin added to ringer lactate, 75 to 100 mL/h for 3 h after surgery
Khing Rushulo [31]	2016	India	60 (30/30)	10 mg/kg of TXA, IV. 10 min before skin incision, over 5 min	10 mL normal saline, IV. 10 min before skin incision, over 5 min	10 IU oxytocin IM + 20 IU oxytocin in a pint of ringer’s lactate for 2 h IV, after delivery of neonate
P. Malathi [32]	2016	India	200 (100/100)	10 mg/kg of TXA, IV. 15–20 min before spinal anesthesia	No treatment	10 IU oxytocin IM + 10 IU oxytocin in 500 mL lactated Ringer solution IV, after cord clamping, over 30 min
Samir Abd Allah Ali [33]	2019	Egypt	200 (100/100)	1 g of TXA in 200 mL normal saline, IV. 20 min before skin incision	No treatment	5 IU oxytocin in 500 mL normal saline IV, after delivery of the neonate, over 30 min
Ayman A. Soliman [34]	2021	Egypt	100 (50/50)	1 g/10 mL TXA, IV. 20 min before skin incision. over 10 min	No treatment	20 IU oxytocin in 500 mL normal saline, IV. after delivery of fetus, over 20–30 min
Noshina Shabir [35]	2019	Pakistan	100 (50/50)	1 g/10 mL TXA in 20 mL of 5% dextrose, IV. 20 min before spinal anesthesia	30 mL of 5% dextrose, IV. 20 min before spinal anesthesia	10 IU oxytocin in 500 mL normal saline, IV. after delivery, over 20–30 min

^a^Data are presented as total number (number in the intervention versus number in the control group)

**Table 2:** Methods of blood loss collection and calculation

Randomized Controlled Trial (RCT)		
**Study**	**Blood loss collection**	**Blood loss calculation**
Ali Movafegh [15]	Blood loss was collected through a suction container, gauze pads and operation sheets	The quantity of blood loss (mL) = (weight of used materials—weight of materials prior to used)/1.05 + (the volume included in the suction container after placental delivery)
H. Abdel-Aleem [16]	Blood loss was collected through a suction container, towels during operation, and through plastic drape after operation	The quantity of intra-operative blood loss (mL) = (weight of wet towels -weight of towels prior to used) × 0.9 + (the volume included in the suction container after placental delivery). The quantity of post-operative blood loss (mL) = weight of used drapes—weight of drapes prior to used) × 0.9
Mehmet B. Sentu ¨rk [17]	Blood loss was collected through operation pads and tampons (with 1 g sensitive scale)	The quantity of blood loss (mL) = (weight of used materials—weight of materials prior to used)/1.05
Anuradha Ghosh [18]	Blood loss was collected through a suction container, operation sheets, gauze pieces and mops (1 g is equivalent to 1 mL)	The quantity of intra-operative blood loss (mL) = (weight of used materials—weight of materials prior to used) + (the volume of blood sucked in suction bottle). The quantity of post-operative blood loss (mL) = weight of used pads—weight of pads prior to used
Haitham Torky [19]	Blood loss was collected through towels	The quantity of blood loss (mL) = weight of used materials—weight of materials prior to used
Abd El-Naser Abd El-Gaber [20]	Blood loss was collected through a suction container, towels, and pads	The quantity of intra-operative blood loss (mL) = (weight of used towels—weight of towels prior to used) + (the volume sucked in the suction bottle after placental delivery). The quantity of post-operative blood loss (mL) = weight of used pads—weight of pads prior to used
Forozan Milani [21]	Blood loss was collected through a suction container, gauzes, sterile drapes, and pads	The quantity of intra-operative blood loss (mL) = (weight of used materials—weight of materials prior to used) + (the volume of blood sucked in suction bottle). The quantity of post-operative blood loss (mL) = weight of used pads—weight of pads prior to used
Zahra Naeiji [22]	Blood loss was collected through a suction container, drapes, mops, sponges, pads, and operation table perineal sheet	The quantity of intra-operative blood loss (mL) = (weight of used materials—weight of materials prior to surgery) + (the volume sucked in the suction bottle after placental delivery). The quantity of post-operative blood loss (mL) = weight of used pads—weight of pads prior to used
Amr H. Yehia [23]	Blood loss was collected through a suction container, towels, and pads. Soaked towel = 150 mL. while semi-soaked towel = 75 mL. soaked pads = 50 mL	The quantity of intra-operative blood loss (mL) = (weight of used materials—weight of materials prior to used) + (the volume of blood sucked in suction bottle). The quantity of post-operative blood loss (mL) = weight of used materials—weight of materials prior to used
**Cohort Studies**		
Upasana Goswami [24]	Blood loss was collected through a suction container, and soaked material such as sponges, mops, pads, and drapes were weighed	The quantity of intra-operative blood loss (mL) = (weight of the abdominal swabs and drapes—weight of materials prior to surgery) + (the volume in the suction bottle after placental delivery)
Ming-ying Gai [25]	Blood loss was collected through a suction container, and soaked gauze, pads and a specially designed operation table sheet were weighed	The quantity of blood loss (mL) = (weight of used materials + unused materials—weight of all materials prior to surgery)/1.05 + (the volume included in the suction container after placental delivery)
Tullika Singh [26]	Blood loss was collected through a suction container, sponges, and pads	The quantity of intra-operative blood loss (mL) = (weight of used materials—weight of materials prior to surgery) + (the volume of blood sucked in suction bottle). The quantity of post-operative blood loss (mL) = weight of used pads—weight of pads prior to used
A. V. Chandak [27]	Blood loss was collected through a suction container, pads	The quantity of intra-operative blood loss (mL) = (weight of used materials—weight of materials prior to surgery) + (the volume of blood sucked in suction bottle). The quantity of post-operative blood loss (mL) = weight of used pads—weight of pads prior to used
Leila Sekhavat [28]	Blood loss was measured by weighting-soaked sheet, via a specially designed operating sheet and an electronic scale to weigh all the material (with a 1 g deviation range)	The quantity of intra-operative blood loss (mL) = (weight of used materials–weight of materials prior to surgery)/1.05. The quantity of post-operative blood loss (mL) = weight of used sheets—weight of sheets prior to used
Irene Ray [29]	Blood loss was collected through a suction container, sheets, mops, and pads	The quantity of intra-operative blood loss (mL) = (weight of used materials—weight of materials prior to used)/1.05 + (the volume of blood sucked in suction bottle). The quantity of post-operative blood loss (mL) = weight of used pads—weight of pads prior to used
S.J. Dhivya Lakshmi [30]	Blood loss was collected through a suction container, operation table perineal sheet and mops	The quantity of blood loss (mL) = (weight of used materials—weight of materials prior to surgery) + (the volume of blood in suction container after delivery of placental)
Khing Rushulo [31]	Blood loss was collected through a suction container, pads	The quantity of intra-operative blood loss (mL) = (weight of used materials—weight of materials prior to surgery) + (the volume of blood sucked in suction bottle). The quantity of post-operative blood loss (mL) = weight of used pads—weight of pads prior to used
P. Malathi [32]	Blood loss was collected through a suction container, mops, surgical swabs, and linen	The quantity of intra-operative blood loss (mL) = (weight of used materials—weight of materials prior to used) + (the volume of blood sucked in suction bottle)
Samir Abd Allah Ali [33]	Blood loss was collected through a suction container, towels, and pads	The quantity of intra-operative blood loss (mL) = (weight of used towels—weight of towels prior to surgery) × 0.962 + (the volume of blood sucked in suction bottle). The quantity of post-operative blood loss (mL) = (weight of used pads—weight of pads prior to used) × 0.962
Ayman A. Soliman [34]	Blood loss was collected through sheets, pads, and gauzes	The quantity of blood loss (mL) = (weight of used materials—weight of materials prior to used)/1.06
Noshina Shabir [35]	Blood loss was collected through a suction container, operation sheets and mops	The quantity of intra-operative blood loss (mL) = (weight of used materials—weight of materials prior to used)/1.05 + (the volume of blood sucked in suction bottle). The quantity of post-operative blood loss (mL) = (weight of used materials—weight of materials prior to used)/1.05

**Table 3:** The general characteristics of included studies

Randomized Controlled Trial (RCT)				
**Study**	**Age (years)**^**a**^	**Gestational age (weeks)**^**a**^	**BMI (kg/m**^**2**^**)**^**a**^	**Birth weight (g)**^**a**^
Ali Movafegh [15]	27.0 ± 3.4/27.6 ± 4.1	38.9 ± 0.4/39.0 ± 0.6		
H. Abdel-Aleem [16]	26.34 ± 5.16/26.62 ± 5.05	39.32 ± 1.15/39.31 ± 1.17		3188.47 ± 458.20/3199.18 ± 444.54
Mehmet B. Sentu ¨rk [17]	30.20 ± 6.83/29.22 ± 6.93			
Anuradha Ghosh [18]	25.94 ± 3.78/26.04 ± 3.39	38.62 ± 0.779/38.72 ± 0.671		
Haitham Torky [19]	30.7 ± 4.66/30.8 ± 4.37		26.87 ± 6.19/27.17 ± 5.83	
Abd El-Naser Abd El-Gaber [20]	27.14 ± 4.986/26.77 ± 4.942	38.32 ± 1.124/38.24 ± 1.518	32.88 ± 2.76/33.59 ± 3.22	
Forozan Milani [21]	29.33 ± 5.59/31.2 ± 5.53	37.93 ± 0.69/37.86 ± 0.81		
Zahra Naeiji [22]	27.16 ± 4.64/27.89 ± 4.44	38.70 ± 2.66/38.50 ± 2.83	29.34 ± 2.11/28.58 ± 2.79	3207.40 ± 459.70/3218.30 ± 423.79
Amr H. Yehia [23]	28.4 ± 4.9/28.6 ± 4.7	39.1 ± 1.1/39.0 ± 1.2	27.2 ± 1.6/27.5 ± 2.0	
**Cohort Studies**				
Upasana Goswami [24]	23.6 ± 2.5/24.3 ± 2.6		22.4 ± 1.6/22.8 ± 1.6	
Ming-ying Gai [25]	29.71 ± 4.18/29.75 ± 4.01	38.80 ± 1.11/38.67 ± 1.03		
Tullika Singh [26]	25 ± 1.46/30 ± 1.24	39.1 ± 1.24/39.3 ± 1.28		
A. V. Chandak [27]	24.3 ± 2.6/23.6 ± 2.5			
Leila Sekhavat [28]	26.2 ± 4.7/27.1 ± 4.1			
Irene Ray [29]	25.00 ± 4.71/25.88 ± 5.39	38.92 ± 1.38/39.02 ± 1.42		
S.J. Dhivya Lakshmi [30]	26.77 ± 2.807/26.82 ± 2.801		29.38 ± 1.2/29.12 ± 2.3	
Khing Rushulo [31]	26.6 ± 5.0/28.8 ± 4.7			
P. Malathi [32]	23.40 ± 3.06/23.59 ± 3.56			
Samir Abd Allah Ali [33]	27.81 ± 5.07/28.32 ± 4.65	38.19 ± 0.7/38.22 ± 1.1	29.24 ± 3/29.55 ± 3.08	
Ayman A. Soliman [34]	21.46 ± 2.71/21.46 ± 2.71	39.34 ± 0.47/39.28 ± 0.45	29.01 ± 2.23/28.22 ± 2.06	
Noshina Shabir [35]	26.01 ± 4.69/26.79 ± 5.39	37.95 ± 1.41/38.97 ± 1.44		

^a^ Values are given as mean ± standard deviation, unless indicated otherwise

Data are presented as total number (number in the TXA versus number in the control group)

### Risk of bias

**Fig. 2:**
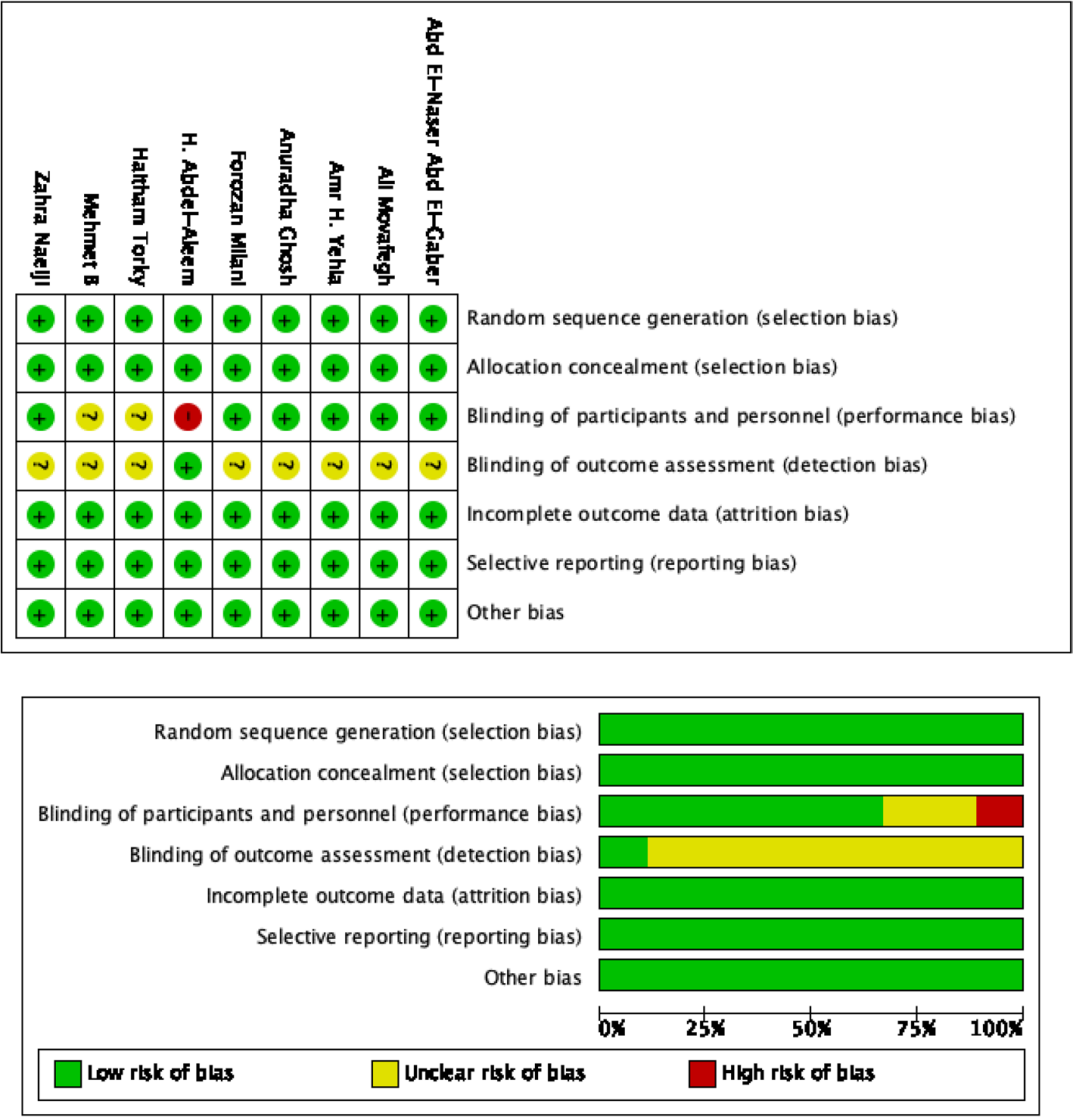
Risk of bias summary and graph showing authors’ judgements about each risk of bias item for RCTs

The result of the quality assessment is presented in Fig. 2 and Table 4. The risk of bias assessment showed that the overall quality of the nine RCTs included are reasonable. The most common bias is detection bias (blinding of outcome assessment). Only one RCT study clearly pointed out that a blinding method was used for outcome assessment, whereas the other trials did not describe the process. This limitation is not easy to overcome in clinical work. For performance bias, one study showed that there was no blinding of participants and personnel, while there is insufficient information in two studies to determine whether there are any such biases. The risk of bias is low for selection bias (random sequence generation and allocation concealment), attrition bias (incomplete outcome data), reporting bias (selective reporting), and other bias. All 12 cohort studies have NOS quality scores of five and above, indicating that these studies are of high quality and can be included in this meta-analysis.

**Table 4:** Results of quality assessment using the Newcastle–Ottawa Scale for cohort studies

Study	Selection				Comparability	Outcome			Quality score
	Representativeness of the exposed cohort	Selection of the non-exposed cohort	Ascertainment of exposure	Demonstration that outcome of interest was not present at start of study	Comparability of cohorts on the bias of the design or analysis^a^	Assessment of outcome	Was follow-up long enough for outcomes to occur	Adequacy of follow-up of cohorts	
Upasana Goswami [24]	☆	☆	☆	☆	☆		☆	☆	7
Ming-ying Gai [25]	☆	☆	☆	☆	☆	☆	☆	☆	8
Tullika Singh [26]	☆	☆	☆	☆	☆		☆	☆	7
A. V. Chandak [27]	☆	☆	☆	☆	☆		☆	☆	7
Leila Sekhavat [28]	☆	☆	☆	☆	☆	☆	☆	☆	8
Irene Ray [29]	☆	☆		☆	☆		☆	☆	6
S.J. Dhivya Lakshmi [30]	☆	☆	☆	☆	☆		☆	☆	7
Khing Rushulo [31]	☆	☆		☆	☆		☆	☆	6
P. Malathi [32]	☆	☆		☆	☆	☆	☆	☆	7
Samir Abd Allah Ali [33]	☆	☆	☆	☆	☆		☆	☆	7
Ayman A. Soliman [34]	☆	☆	☆	☆	☆		☆	☆	7
Noshina Shabir [35]	☆	☆	☆	☆	☆		☆	☆	7

^a^A maximum of 2 stars can be assigned in this category

### Primary outcomes

#### Blood loss during cesarean section

**Fig. 3:**
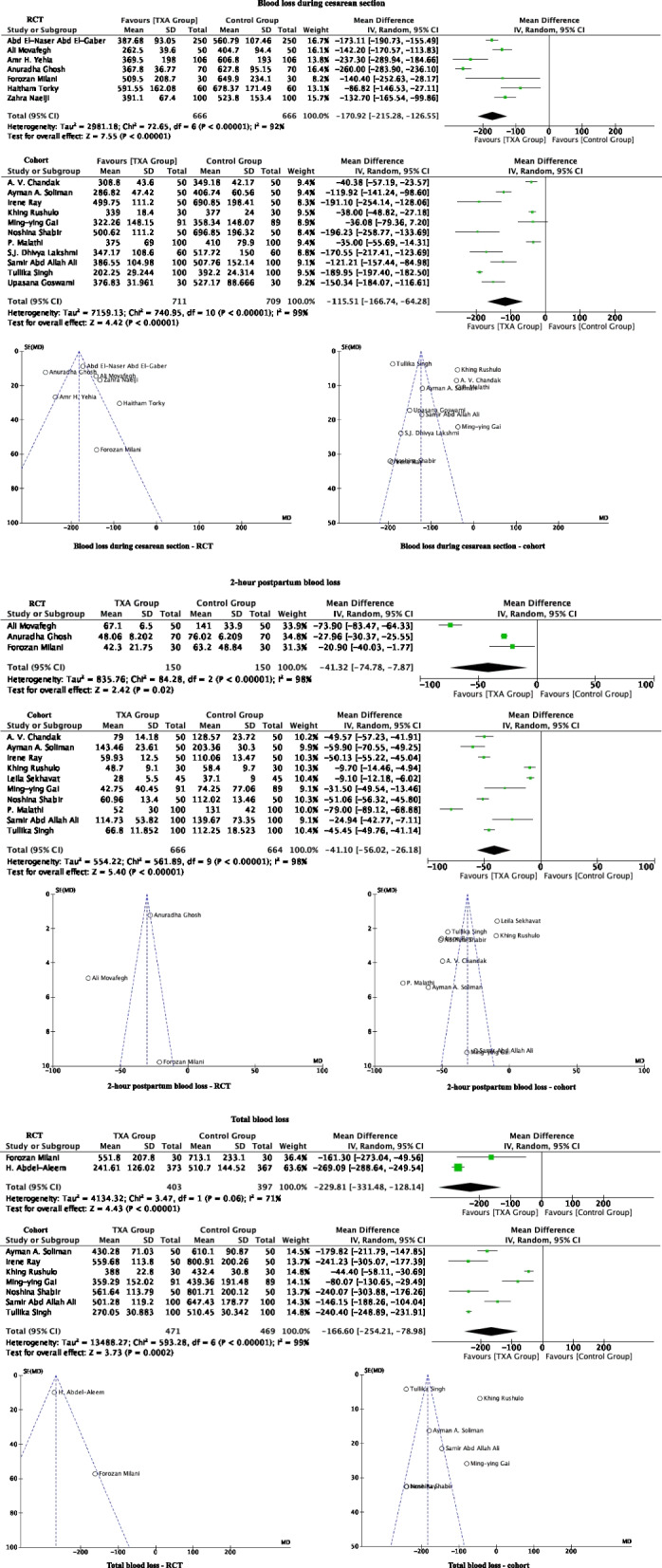
Forest plot diagram showing the effect of prophylactic TXA use before cesarean section on intraoperative blood loss, 2-h postpartum blood loss and total blood loss

There are 18 studies (seven RCTs and 11 cohort studies) with a total of 2752 patients, where 1377 were in the TXA group and 1375 in the control group. The mean intraoperative blood loss in the TXA group was less than that of the control group. This reduction in blood loss during operation with TXA administration is significant in both the RCT studies (seven studies with 666 TXA patients and 666 controls; MD: -170.92 mL; 95% CI: -215.28, -126.55; *P* < 0.00001) and the cohort studies (11 studies with 711 TXA patients and 709 controls; MD: -115.51 mL; 95% CI: -166.74, -64.28; *P* < 0.00001) (Fig. 3 and Table 5).

**Table 5:** Postpartum hemorrhage related outcomes

Randomized Controlled Trial (RCT)										
**Study**	**Blood Loss (mL)**^**a**^		**Blood Loss (mL)**^**b**^		**Blood Loss (mL)**^**c**^		**Blood Transfusion (n)**		**Hemoglobin Change (g/dL)**	
	**TXA**	**Control**	**TXA**	**Control**	**TXA**	**Control**	**TXA**	**Control**	**TXA**	**Control**
Ali Movafegh[15]	262.5 ± 39.6	404.7 ± 94.4	67.1 ± 6.5	141.0 ± 33.9					1.0 ± 0.4	1.8 ± 0.7
H. Abdel-Aleem[16]					241.61 ± 126.02	510.70 ± 144.52			0.48 ± 0.87	1.42 ± 1.16
Mehmet B. Sentu ¨rk									1.11 ± 0.62	1.27 ± 0.66
Anuradha Ghosh	367.8 ± 36.77	627.8 ± 95.15	48.06 ± 8.202	76.02 ± 6.209					1.16 ± 0.316	1.83 ± 0.372
Haitham Torky	591.55 ± 162.08	678.37 ± 171.49					3/60	9/60		
Abd El-Naser Abd El-Gaber	387.68 ± 93.05	560.79 ± 107.46	159.16 ± 50.95*	232.68 ± 65.18*	546.84 ± 106.13^**#**^	793.99 ± 141.21^**#**^			0.475 ± 0.23	1.06 ± 0.31
Forozan Milani	509.5 ± 208.7	649.9 ± 234.1	42.3 ± 21.75	63.2 ± 48.84	551.8 ± 207.8	713.1 ± 233.1				
Zahra Naeiji	391.1 ± 67.4	523.8 ± 153.4	56.0 ± 21.9*	61.6 ± 30.6*			5/100	13/100		
Amr H. Yehia	369.5 ± 198	606.8 ± 193	85.0 ± 30.7*	130.8 ± 49.3*	454.5 ± 201^**#**^	737.6 ± 217^**#**^				
**Cohort Studies**										
Upasana Goswami[24]	376.83 ± 31.961	527.17 ± 88.666								
Ming-ying Gai[25]	322.26 ± 148.15	358.34 ± 148.07	42.75 ± 40.45	74.25 ± 77.06	359.29 ± 152.02	439.36 ± 191.48				
Tullika Singh	202.25 ± 29.244	392.20 ± 24.314	66.80 ± 11.852	112.25 ± 18.523	270.05 ± 30.883	510.45 ± 30.342				
A. V. Chandak	308.8 ± 43.6	349.18 ± 42.17	79.0 ± 14.18	128.57 ± 23.72						
Leila Sekhavat			28.0 ± 5.5	37.1 ± 9.0					0.1 ± 0.6	2.5 ± 0.8
Irene Ray	499.75 ± 111.20	690.85 ± 198.41	59.93 ± 12.5	110.06 ± 13.47	559.68 ± 113.80	800.91 ± 200.26			0.26 ± 0.22	0.99 ± 0.48
S.J. Dhivya Lakshmi	347.17 ± 108.6	517.72 ± 150								
Khing Rushulo	339.0 ± 18.4	377 ± 24.0	48.7 ± 9.1	58.4. ± 9.7	388.0 ± 22.8	432.4 ± 30.8			0.52 ± 0.2	0.84 ± 0.2
P. Malathi	375 ± 69	410 ± 79.9	52 ± 30	131 ± 42					1.34 ± 0.15	1.44 ± 0.88
Samir Abd Allah Ali	386.55 ± 104.98	507.76 ± 152.14	114.73 ± 53.82	139.67 ± 73.35	501.28 ± 119.2	647.43 ± 178.77	0/100	1/100	0.73 ± 0.58	1.35 ± 0.56
Ayman A. Soliman	286.82 ± 47.42	406.74 ± 60.56	143.46 ± 23.61	203.36 ± 30.30	430.28 ± 71.03	610.10 ± 90.87				
Noshina Shabir	500.62 ± 111.20	696.85 ± 196.32	60.96 ± 13.4	112.02 ± 13.46	561.64 ± 113.79	801.71 ± 200.12				

Values are given as mean ± standard deviation

^a^ blood loss from placental delivery to the end of CS (mL)

^b^ blood loss from end of CS to 2 h postpartum (mL)

^c^ blood loss from placental delivery to 2 h postpartum (mL)

^*^ blood loss from end of CS to 6 h postpartum (mL)

^**#**^ blood loss from placental delivery to 6 h postpartum (mL)

#### 2-h postpartum blood loss

Thirteen studies (three RCTs and 10 cohort studies) reported the 2-h postpartum blood loss for a total of 1630 patients (816 TXA and 814 controls). The TXA group showed reduction of blood loss compared to control. This trend remained significant when the RCT (three studies with 150 TXA patients and 150 controls; MD:—41.32 mL; 95% CI: -74.78, -7.87; *P* = 0.02) and cohort studies (10 studies with 666 TXA patients and 664 controls; MD: -41.10 mL; 95% CI: -56.02, -26.18; *P* < 0.00001) were analyzed separately. (Fig. 3 and Table 5).

#### Total blood loss (during cesarean section plus 2-h postpartum)

Total blood loss was reported in 10 studies (two RCTs and eight cohort studies) for a total of 1740 patients (874 TXA and 866 controls). The mean total blood loss was lower in the TXA group compared to control. The trend again remained significant when the RCT (two studies with 403 TXA patients and 397 controls; MD: -229.81 mL; 95% CI: -331.48, -128.14; *P* < 0.00001) and cohort studies (eight studies with 471 TXA patients and 469 controls; MD: -166.60 mL; 95% CI: -254.21, -78.98; *P* = 0.0002) were analyzed separately (Fig. 3 and Table 5).

### Secondary outcomes

#### 6-h postpartum blood loss

**Fig. 4:**
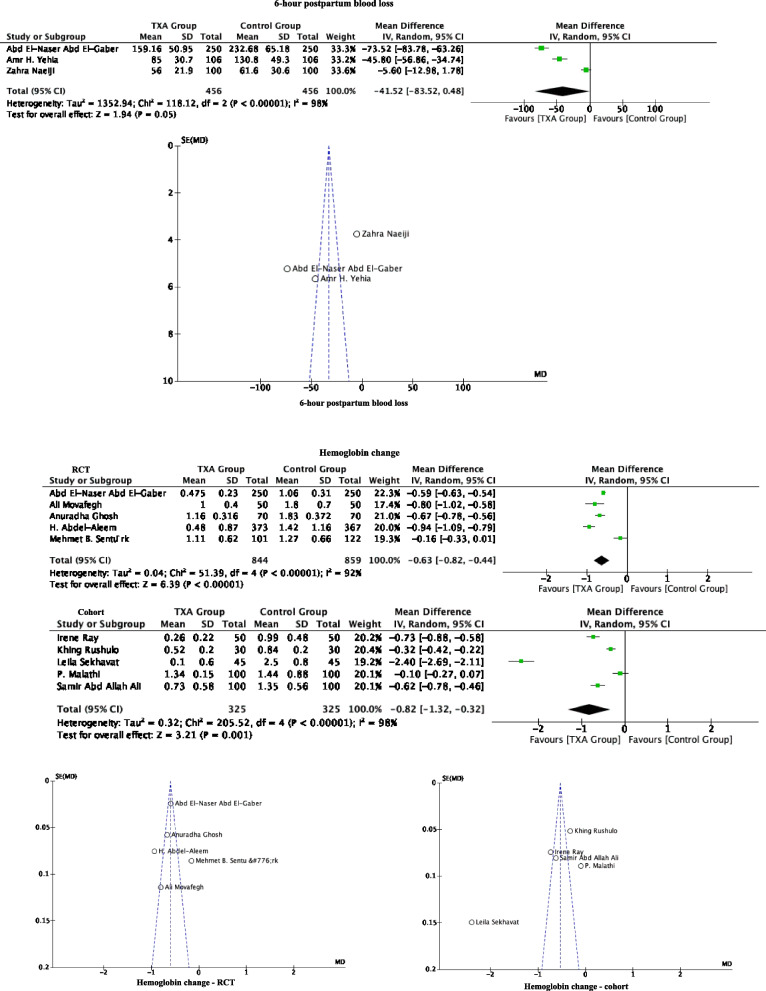
Forest plot diagram showing the effect of prophylactic TXA use before cesarean section on 6-h postpartum blood loss and hemoglobin change

The 6-h postpartum blood loss was only reported in three RCT studies, which involved a total of 912 patients (456 TXA and 456 controls). These studies showed that the TXA group has a mean reduction of 41.52 mL (95% CI: -83.52, 0.48) of blood loss compared to the control group, but this was not statistically significant (*P* = 0.05) (Fig. 4 and Table 5).

#### Hemoglobin change

Ten studies (five RCTs and 5 cohort studies) documented hemoglobin changes. A total of 2353 patients were involved, with1169 in the TXA group and 1184 in the control group. Compared with the control group, prophylactic TXA reduced the hemoglobin. Significant differences were also observed when the RCT (five studies with 844 TXA patients and 859 controls; MD:—0.63 g/dL; 95% CI: -0.82, -0.44; *P* < 0.00001) and cohort studies (five studies with 325 TXA patients and 325 controls; MD:—0.82 g/dL; 95% CI: -1.32, -0.32; *P* = 0.0001) were analyzed separately (Fig. 4 and Table 5).

### Sensitivity analysis

A “leave-one-out” sensitivity analysis showed that our results were robust and individual elimination of each of the included studies did not cause any substantial variation in our findings.

## Discussion

Maternal mortality is an important concern in both developed and developing countries, and PPH is a major contributor. Especially in low-income countries, PPH is the number one cause of maternal death. Current data suggests that one woman dies from postpartum hemorrhage every seven minutes worldwide [36]. Thus, efforts to reduce maternal mortality must include a focus on the prevention of PPH. Since 2018, the World Health Organization (WHO) [37] has recommended that women undergoing delivery, whether by cesarean section or vaginally, should be given uterotonic agents to prevent the occurrence of PPH. However, PPH remains a very common morbidity. At present, oxytocin is the standard drug for the prevention of PPH in the clinic, but it has a short half-life and requires continuous use to sustainably contract the uterus. However, repeated use can lead to receptor desensitization, which reduces the effectiveness of oxytocin. Some patients may also experience hypertension, water and sodium retention and other adverse effects. A 2018 report noted that the incidence of PPH remains high at 15% even with the prophylactic use of oxytocin [38]. Previous studies [39] also showed that women who delivered by cesarean section have significantly higher blood loss than those who delivered by the transvaginal method. Therefore, to better prevent the occurrence of PPH in cesarean section patients, there is a clinical preference to adopt a combined medication regimen.

Antifibrinolytics are common drugs for the prevention and treatment of bleeding by competitively inhibiting the binding of plasminogen to fibrin, thus affecting the body's endogenous hemostatic process, and reducing excessive bleeding. TXA is one such drug that is more commonly used in the clinics as it has good records of reducing blood lost in different circumstances. A worldwide study of 130,000 traumatic brain injury patients by the CRASH-3 trial collaborators [40], showed that the administration of TXA within three hours of head injury significantly reduces the occurrence of intracranial hemorrhage and mortality. Similarly, a RCT study in 2016 [41] showed that prophylactic administration of TXA significantly reduces the incidence of bleeding after benign hysterectomy and reoperation for postoperative bleeding. These studies demonstrate the potential of TXA to reduce bleeding.

In this systematic review and meta-analysis, we included randomized controlled trials and cohort studies on the effect of preoperative prophylactic use of TXA on perioperative blood loss in cesarean sectioned women, including hemoglobin changes and blood losses intraoperatively, 2-h postpartum, total (during cesarean section and 2-h postpartum) and 6-h postpartum. We found that prophylactic use of TXA before cesarean section significantly reduced the intraoperative blood loss (RCT: -170.92 mL; 95% CI: -215.28, -126.55; Cohort: -115.51 mL; 95% CI: -166.74, -64.28), 2-h postpartum blood loss (RCT: -41.32 ml; 95% CI: -74.78, -7.87; Cohort: -41.1 mL; 95% CI: -56.02, -26.18) and total blood loss (RCT: -229.81 ml; 95% CI: -331.48, -128.14; Cohort: -166.6 mL; 95% CI: -254.21, -78.98), and hemoglobin change (RCT: -0.63 g/dL; 95% CI: -0.82, -0.44; Cohort: -0.82 g/dL; 95% CI: -1.32, -0.32) compared with the control group. While the 6-h postoperative blood loss was also reduced in the TXA group, it did not reach statistical significance.

In recent years, the role of TXA in reducing PPH has gradually been appreciated. He et al.[42] simulated antithrombotic therapy in vitro by adding a thrombin inhibitor in the presence of low concentration of TXA (0.4 mg/L), which increased the clot lysis time from 40 to 50 min. In the presence of FXa inhibitors, the dissolution time increased from 25 to 50 min. However, when TXA was administered at a higher dose of 9.5 mg/L, fibrinolysis was abolished in both the thrombin and FXa inhibitor groups. In support of this, an in vitro study from 1968 showed significant antifibrinolytic activity when TXA was used at 10 mg/L or more [43]. Subsequently, a metabolic study by Andersson et al. [44] showed that in healthy adults, intravenous administration of 10 mg/kg TXA achieved blood concentrations of approximately 18 µg/mL after 1 h, and declined to approximately 10 µg/mL after 3 h and 5 µg/mL after 5 h. Thus, 55% of TXA was already metabolized via the kidneys three hours after administration, and 90% was metabolized after 24 h, giving TXA a half-life of approximately three hours. This may explain the finding in our study that blood loss six hours after prophylactic use of TXA was not significantly different from that of the control group.

An in-depth research of cesarean deliveries by Gilliot et al. [45] showed that 90% of patients given 1 g TXA attained blood concentration of TXA ≥ 30 mg/L during the first 15 min after administration. According to the pharmacokinetic characteristics of TXA, preoperative prophylactic administration of TXA may play a role before activation of the fibrinolytic system, thereby reducing the occurrence of hemorrhage. TXA has been used to treat severe PPH and save maternal lives since 1972 [46]. The woman trial [9] in 2019 involving 20,021 women showed a significant reduction in the incidence and mortality of maternal hemorrhage when TXA was administered within three hours of delivery, and highlighted the importance of early administration. Despite these evidence for the benefit of TXA in PPH and other circumstances where bleeding is a risk, there is currently no consensus guideline for the prophylactic use of TXA in cesarean section patients. In this study, RCT and cohort studies, analyzed separately, showed consistently that preoperative prophylactic intravenous administration of TXA is beneficial in reducing intra- and postoperative bleeding.

A study shows that patients who use TXA in hip and knee arthroplasty have no increased risk of vascular occlusion events [47]. Although there is research evidence [48] shows that TXA can reduce blood transfusion during surgery, there is still uncertainty whether TXA is related to the increased risk of arterial and venous thromboembolism, which limits its wide application. Currently, the role of TXA in preventing bleeding and the impact of thromboembolic events require further attention, and high-quality studies with more sample sizes are still needed for further in-depth exploration.

## Data Availability

The original contributions presented in the study are included in the article/supplementary material, further inquiries can be directed to the corresponding author/s.

## Abbreviations

CI: Confidence interval
PPH: Postpartum hemorrhage
RR: Relative risk
TXA: Tranexamic acid
